# Air pollution after acute bronchiolitis is a risk factor for preschool asthma: a nested case-control study

**DOI:** 10.1186/s12940-023-01035-1

**Published:** 2023-12-04

**Authors:** Hao-Wei Chung, Hui-Min Hsieh, Chung-Hsiang Lee, Yi-Ching Lin, Yu-Hsiang Tsao, Ming-Chu Feng, Chih-Hsing Hung

**Affiliations:** 1 Department of Pediatrics, Kaohsiung Medical University Chung Ho Memorial Hospital, Kaohsiung Medical University, Kaohsiung, Taiwan; 2 Department of Biological Science and Technology, National Yang Ming Chiao-Tung University, Hsinchu, Taiwan; 3 Department of Pediatrics, Kaohsiung Municipal Siaogang Hospital, Kaohsiung, Taiwan; 4 Department of Public Health, Kaohsiung Medical University Chung Ho Memorial Hospital, Kaohsiung Medical University, Kaohsiung, Taiwan; 5 Department of Medical Research, Kaohsiung Medical University Chung Ho Memorial Hospital, Kaohsiung Medical University, Kaohsiung, Taiwan; 6 Department of Community Medicine, Kaohsiung Medical University Chung Ho Memorial Hospital, Kaohsiung Medical University, Kaohsiung, Taiwan; 7 Center for Big Data Research, Kaohsiung Medical University, Kaohsiung, Taiwan; 8 Department of Laboratory Medicine, Kaohsiung Medical University Chung Ho Memorial Hospital, Kaohsiung Medical University, Kaohsiung, Taiwan; 9 Doctoral Degree Program in Toxicology, College of Pharmacy, Kaohsiung Medical University, Kaohsiung, Taiwan; 10 Department of Laboratory Medicine, School of Medicine, College of Medicine, Kaohsiung Medical University, Kaohsiung, Taiwan; 11 Department of Medical Research, Kaohsiung Medical University Chung Ho Memorial Hospital, Kaohsiung, Medical University, Kaohsiung, Taiwan; 12 Department of Dysphagia Functional Reconstructive Center, Kaohsiung Municipal Siaogang Hospital, Kaohsiung, Taiwan; 13 Department of Nursing, Fooyin University, Kaohsiung, Taiwan; 14 Research Center for Environmental Medicine, Kaohsiung Medical University, Kaohsiung, Taiwan; 15 Department of Pediatrics, Faculty of Pediatrics, College of Medicine, Kaohsiung Medical University, Kaohsiung, Taiwan

**Keywords:** Ambient air pollution, Preschool asthma, Acute bronchiolitis infant

## Abstract

**Background:**

Acute bronchiolitis and air pollution are both risk factor of pediatric asthma. This study aimed to assess subsequent exposure to air pollutants related to the inception of preschool asthma in infants with acute bronchiolitis. This study aimed to assess subsequent exposure to air pollutants related to the inception of preschool asthma in infants with acute bronchiolitis.

**Methods:**

A nested case-control retrospective study was performed at the Kaohsiung Medical University Hospital systems between 2009 and 2019. The average concentration of PM_10_, PM_2.5_, SO_2_, NO, NO_2,_ and NO_X_ was collected for three, six, and twelve months after the first infected episode. Adjusted regression models were employed to evaluate the association between asthma and air pollution exposure after bronchiolitis.

**Results:**

Two thousand six hundred thirty-seven children with acute bronchiolitis were included. Exposure to PM_10_, PM_2.5_, SO_2_, NO, NO_2,_ and NO_X_ in the three, six, and twelve months following an episode of bronchiolitis was found to significantly increase the risk of preschool asthma in infants with a history of bronchiolitis.(OR, 95%CI: PM_10_ = 1.517-1.559, 1.354–1.744; PM_2.5_ = 2.510-2.603, 2.148–3.061; SO_2_ = 1.970-2.040, 1.724–2.342; ; NO = 1.915-1.950, 1.647–2.272; NO_2_ = 1.915-1.950, 1.647–2.272; NO_X_ = 1.752-1.970, 1.508–2.252) In a sensitive analysis of hospitalized infants, only PM_10_, PM_2.5_, SO_2,_ and NO were found to have significant effects during all time periods. (OR, 95%CI: PM_10_ = 1.613-1.650, 1.240–2.140; PM_2.5_ = 2.208-2.286, 1.568–3.061; SO_2_ = 1.679-1.622, 1.197–2.292; NO = 1.525-1.557, 1.094–2.181)

**Conclusion:**

The presence of ambient PM_10_, PM_2.5_, SO_2_ and NO in the three, six, and twelve months following an episode of acute bronchiolitis has been linked to the development of preschool asthma in infants with a history of acute bronchiolitis.

**Supplementary Information:**

The online version contains supplementary material available at 10.1186/s12940-023-01035-1.

## Introduction

For children, air pollutions are implicated in vulnerability of acute lung infection and the development of chronic lung inflammation [1–3]. Acute bronchiolitis, the most common lower tract infection under the age of two, would not only cause respiratory distress impeding appropriate oral intake but increase risk for development of pediatric asthma [4]. Meta analysis concluded that children who acquired acute bronchiolitis before two years of age had higher risk to incept the wheezing/asthma in later life [5]. Acute bronchiolitis is the leading cause of frequent clinician visits or hospitalization of children under two years of age [6]. Therefore, most studies implicated that ambient air pollutions exposure such as particulate matter of 2.5 mm in diameter (PM_2.5_), particulate matter of 10 mm in diameter (PM_10_), and nitrogen dioxide (NO_2_) were associated with increase of acute bronchiolitis hospitalization and severity [3, 7, 8].

Asthma is the most common chronic, noncommunicable respiratory diseases among the children worldwide [9]. Pediatric asthma lead to not only directed medical cost such as high-cost emergency care and hospitalizations but indirect non-medical cost such as time lost from school, declining productivity at school [9, 10]. Since 1950, the pediatric asthma prevalence ascended apparently around the world, and it is expected to reach 400 million people by 2025 [11]. Emerging evidence from epidemiological studies indicates that air pollutions are associated with new onset asthma in the pediatric population, whereas such associations are less clear in adults [12–14]. Asthma involved a complex interaction between the genetics, drugs, environment, and infection [15]. Viral respiratory infection would change the lung development and possibly make the child more vulnerable to subsequent exposure to air pollutions [16]. However, few studies focus on the role of air pollutants as a risk factor for infant to incept asthma after bronchiolitis.

Therefore, we speculated that the oxidative stress caused by air pollutions might be different in the development of asthma for different severity of acute bronchiolitis. For further primary prevention strategy in these high-risk infants, the aim of this study is to assess the relationship of air pollutions exposure after acute bronchiolitis and new-onset preschool asthma.

## Material and methods

### Study population, data source, and procedure

This retrospective nested case–control study was extracted from electronic medical records (EMRs) in the Kaohsiung Medical University Hospital Research Database (KMUHRD). The KMUHRD, more than three million EMRs with patient identifiers from multiple hospitals within the KMU health system (one tertiary medical center, two regional hospitals and one local hospital) in Kaohsiung city, southern Taiwan, had been used for pediatrics population study [17]. Using KMUHRD EMR record data from 2009 to 2019, we first identified children with first diagnosis of acute bronchiolitis before 24 months old at the outpatient visit or inpatient admission who remained in our health system at least five years after index date. We used narrow definition of acute bronchiolitis as previous study [18]. (International Classification of Diseases, Ninth Revision, Clinical Modification [ICD-9-CM] code: 466, 466.0, 466.1, 466.11, 466.19, and 490 or ICD-10 CM code: J20.8, J20.9,J21.0-9, and J40 with an age limited) The case group included children who had acute bronchiolitis and developed asthma, and the control group included children who had acute bronchiolitis without any medical history of asthma. We included children aged 2 to 6 years diagnosed with preschool asthma, defined by either ICD-9 CM code 493 or ICD-10 CM code J45, coupled with a requirement of at least two prescriptions for antiasthma drugs at different times within a two-year period [19]. Antiasthma drugs included inhaled selective b2-agonists (ATC code R03AC), inhaled corticosteroids (ATC code R03BA), combined inhaled salbutamol/sodium cromoglycate (ATC code R03AK04), and combined inhaled selective b2-agonists/corticosteroids (ATC code R03AK06, R03AK07).

**Fig. 1:**
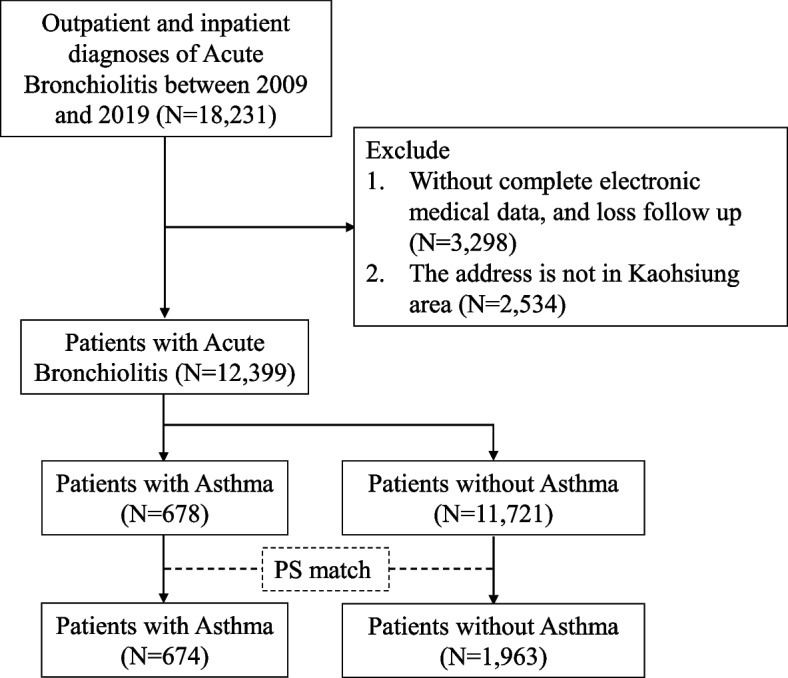
The flowchart of the inclusion and exclusion criteria

The date of the first asthma diagnosis was defined as index date in the case group and the same index date was applied to the matched control child without asthma. We used a 1-to-2 propensity score matching (PSM) approach to match cases and control patients based on their propensity scores, which were generated using logistic regression with the following covariates: sex, index age group, and comorbidities. In PSM, units are paired based on similar propensity scores, with unmatched units being discarded. This approach helps to reduce bias by balancing the distribution of covariates between the treated and untreated groups [20]. The flowchart of the inclusion and exclusion criteria is shown in Fig. 1.

### Ethical considerations

The data from KMUHRD followed the 1964 Declaration of Helsinki and the Personal Information Protection Act were without any personal identification information. The data were analyzed from February to October 2022. The Institutional Review Board of the KMUH (IRB number: KMUHIRB-E(I)-20200002) approved this study and waived the requirement for written informed consent because of the retrospective design and use of deidentified data.

### Data collection and classification

In addition to the covariates of age and sex, potential baseline confounders known to be associated with asthma were controlled including birthweight, perinatal infection [ICD-9-CM code 7602, ICD-10-CM code P399], allergic rhinitis [ICD9-CM code 477, ICD10-CM code J30], acute sinusitis [ICD9-CM code 4619, ICD-10-CM code J01], chronic sinusitis [ICD9-CM code 4739, ICD10-CM code J32], and atopic dermatitis [ICD-9-CM code 691.8, ICD10-CM code L2089, L209]). In our database, we were unable to reach family history of atopy and asthma, especially maternal asthma, and exposure to tobacco smoking. Subgroup analysis was basing on age of first acute bronchiolitis, hospitalization for acute bronchiolitis, and total IgE level.

### Particulate matter exposure assessment

Each patient’s address was confirmed with the detail to the district level to match the nearest air quality monitoring station within 10 kilometers. We analyzed monthly average concentrations of longitudinal air quality, including PM_2.5_, PM_10,_ SO_2_, Nitric oxide (NO), NO_2,_ and Nitrogen oxides (NO_X_ ) levels, collected from thirteen air quality monitoring stations around Kaohsiung city. We measured average cumulative air pollution exposure at three, six, and twelve months after first episode of acute bronchiolitis. The cumulative air pollution per interquartile range (IQR) was used to adjust the mean variability among multiple datasets.

### Statistical analysis

All statistical analyses were performed using SAS® software, version 9.4 of the SAS System for Windows (Copyright©2020. SAS Institute Inc. product or service names are registered trademarks or trademarks of SAS Institute Inc., Cary, NC, USA). A p value <0.05 was considered statistically significant.

Chi-square tests were used to evaluate the significance of differences in categorical variables between children with and without asthma. Propensity score analysis was performed through logistic regression in a ratio of 1-to-2 to obtain a matched case with age of acute bronchiolitis, gender, and comorbidity variables. The association between major air pollution exposure and the risk of asthma was analyzed by conditional logistic regression while controlling baseline demographic characteristics (age, sex). Odds ratios (ORs) adjusted ORs (aORs) and 95% confidence intervals (CIs) showed the risk of the development of asthma in different cumulative air pollution exposure periods.

## Results

Before PSM, there were 11,721 children without asthma and 678 children with asthma. Most of the children were female, with 56.39% of those without asthma and 63.42% of those with asthma being female (*p* < 0.001). The birth body weight was significantly different between the two groups, with 3.87% of children without asthma and 6.34% of those with asthma having a birth body weight of less than 2500 g (*p *= 0.011). Prematurity was not significantly different between the two groups. The age of asthma diagnosis was slightly higher in children with asthma compared to those without asthma (4.17±1.28 years vs 4.06±1.17 years, *p* = 0.031). However, the age of acquiring the first bronchiolitis was significantly higher in children with asthma compared to those without asthma (1.10±0.53 years vs 0.97±0.51 years, *p *<0.001). Besides, allergic rhinitis was present in 44.69% of children with asthma and 9.91% of those without asthma before PSM (*p* < 0.001). Atopic dermatitis was present in 17.85% of children with asthma and 10.05% of those without asthma before PSM (*p* <0.001). After PSM, there were 1,963 children without asthma and 674 children with asthma. After PSM, no significant differences were observed in the proportion of gender, low birth weight, prematurity, age of asthma diagnosis, age of acquiring the first bronchiolitis, allergic rhinitis, atopic dermatitis, or chronic sinusitis between the two groups. After PSM, the proportion of hospitalized children was still significantly higher in those with asthma (28.07% vs 42.73%, *p* < 0.001). The demographic and clinical characteristics of children with acute bronchiolitis, with and without asthma, before and after PSM are presented in Table 1. The distributions of each air pollutants in the three, six, and twelve months after acute bronchiolitis are in Table 2.

**Table 1:** Demographic and clinical characteristics in acute bronchiolitis children with/without asthma

	**Before PSM Matching**		*p*-value	**After PSM Matching**		*p*-value
	Without asthma	Asthma		Without asthma	Asthma	
**N**	11,721	678		1,963	674	
**Gender (N, %)**^a^						
Female	6,610(56.39%)	430(63.42%)	<0.001	1,225(62.40%)	428(63.50%)	0.611
Male	5,111(43.61%)	248(36.58%)		738(37.60%)	246(36.50%)	
**Birth body weight (N, %)**^a^						
< 2500 g	454(3.87%)	43(6.34%)	0.011	121(6.16%)	43(6.38%)	0.713
≧2500 g	11,267(96.13%)	635(93.66%)		1,842(93.84%)	631(93.62%)	
**Gestational age (N, %)**^a^						
< 37 weeks	116 (0.99%)	3 (0.44%)	0.515	29(1.47%)	3(0.45%)	0.184
≧ 37 weeks	11,605 (99.01%)	675(99.56%)		1934(98.53%)	671(99.55%)	
**Asthma diagnosis age (Years, Means ± SD)**	4.06±1.17	4.17±1.28	0.031	4.11±1.20	4.17±1.28	0.253
**Age acquired 1st bronchiolitis (Years, Means ±SD)**	0.97±0.51	1.10±0.53	<0.001	1.11±0.52	1.10±0.53	0.647
**Age acquired 1st bronchiolitis**						
< 1 year old (N, %)	6,407(54.66%)	297(43.81%)	<0.001	852(43.40%)	297(44.07%)	0.765
1-2 years old (N, %)	5,314(45.34%)	381(56.19%)		1,111(56.60%)	377(55.93%)	
**Hospitalization for acute bronchiolitis (N, %)**						
No	8443(72.03%)	387(57.08%)	<0.001	1,412(71.93%)	386(57.27%)	<0.001
Yes	3278(27.97%)	291(42.92%)		551(28.07%)	288(42.73%)	
**Total IgE leve**l						
≦100 IU/ml **(N, %)**	770(49.52%)	146(38.73%)	<0.001	188(44.87%)	143(38.24%)	0.059
>100 IU/ml **(N, %)**	785(50.48%)	231(61.27%)		231(55.13%)	231(61.76%)	
**Comorbidity (N, %)**^a^						
Allergic rhinitis	1,162(9.91%)	303(44.69%)	<0.001^a^	840(42.79%)	299(44.36%)	0.478
Atopic dermatitis	1,178(10.05%)	121(17.85%)	<0.001^a^	306(15.59%)	120(17.80%)	0.178
Chronic sinusitis	35( 0.30%)	16(2.36%)	<0.001^a^	23( 2.36%)	12(1.78%)	0.234

Chi-square test was used to test binary or category variables and t-test was used to test continuous variables between children acquired bronchiolitis with or without asthma. A *p*-value below 0.05 was considered statistically significant

*PSM* Propensity score matching

^a^variables used for propensity score matching

**Table 2:** Concentration of subsequent ambient air pollutants in different periods in all infants acquired acute bronchiolitis

Pollutants	Mean ± SD	Min	Percentile			Max	IQR
			25%	50%	75%		
0-3 months							
SO2(ppb)	8.27 ± 2.17	1.67	6.73	8.05	9.79	12.49	3.06
PM_2.5_ (μg/m^3^)	43.74 ± 4.03	13.20	40.92	44.26	46.78	52.03	5.85
PM_10_ (μg/m^3^)	77.30 ± 6.86	25.25	72.96	76.30	81.33	91.10	8.37
NO(ppb)	6.98 ± 2.11	1.62	5.35	6.76	8.77	15.28	3.42
NO2(ppb)	22.19 ± 3.82	2.07	19.75	22.50	25.45	29.81	5.70
NOx(ppb)	29.17 ± 5.85	3.70	25.17	29.38	34.30	43.75	9.14
0-6 months							
SO2(ppb)	8.21 ± 2.15	1.66	6.69	8.01	9.71	12.33	3.02
PM_2.5_ (μg/m^3^)	43.46 ± 4.02	13.02	40.62	44.08	46.65	51.35	6.03
PM_10_ (μg/m^3^)	76.94 ± 6.81	25.06	72.66	76.02	81.04	91.10	8.38
NO(ppb)	6.92 ± 2.09	1.61	5.31	6.70	8.69	15.11	3.39
NO2(ppb)	22.09 ± 3.79	2.07	19.64	22.44	25.39	29.00	5.75
NOx(ppb)	29.01 ± 5.79	3.70	25.03	29.34	34.02	43.42	9.00
0-12 months							
SO2(ppb)	8.09 ± 2.14	1.63	6.53	7.88	9.62	12.20	3.09
PM_2.5_ (μg/m^3^)	43.04 ± 4.25	12.77	39.92	43.74	46.08	51.41	6.16
PM_10_ (μg/m^3^)	76.50 ± 7.08	25.26	72.07	75.79	80.51	91.10	8.44
NO(ppb)	6.82 ± 2.08	1.62	5.21	6.51	8.63	15.10	3.42
NO2(ppb)	22.00 ± 3.80	2.07	19.57	22.48	25.24	28.87	5.66
NOx(ppb)	28.82 ± 5.80	3.70	24.75	29.06	33.80	43.38	9.05

*Abbreviations*: *SD* Standard deviation, *IQR* Interquartile range, *SO*_*2*_ Sulphur dioxide, *PM*_*2.5*_ Particulate matters with diameters at 2.5 micrometers and smaller, *PM*_*10*_ Particulate matters with diameters at 10 micrometers and smaller, *NO* Nitrogen oxide, *NO*_*2*_ Nitrogen dioxide, *NO*_*X*_ Nitrogen oxides, *OR* crude odds ratio, *aOR* Adjusted odds ratio

Table 3 summarizes the results obtained from various conditional logistic regression models that investigated the relationship between different durations of exposure to air pollutants after acute bronchiolitis and the risk of developing asthma in all eligible children. The adjusted odds ratios (aORs) for asthma associated with exposure to each air pollutant (namely, PM_2.5_, PM_10_, SO_2_, NO, NO_2_, and NO_X_) were found to be statistically significant during the three post-infection periods. Notably, exposure to PM_2.5_ demonstrated the strongest association with aORs (95% confidence intervals [CIs]) of 2.510 (2.148-2.935), 2.603 (2.214-3.061), and 2.538 (2.172-2.965) in the three, six, and twelve months following acute bronchiolitis, respectively. Furthermore, SO2 exposure was significantly associated with aORs (95% CIs) of 1.977 (1.728-2.261), 1.970 (1.724-2.252), and 2.040 (1.777-2.342) during the three, six, and twelve months after acute bronchiolitis, respectively. Conversely, PM_10_ exposure was associated with the lowest significant aORs (95% CIs) of 1.52 (1.35–1.70), 1.54 (1.37–1.72), and 1.56 (1.39–1.74) during the three, six, and twelve months following acute bronchiolitis, respectively.

**Table 3:** Associations between subsequent exposure to ambient air pollutants and preschool asthma in acute bronchiolitis infants. (*n*=2637)

Major air pollutions (per IQR)	OR	95%CI	*p*-value	aOR^a^	95%CI	*p*-value
0-3 months						
SO_2_ (ppb)	1.978	1.730-2.262	<.0001	1.977	1.728-2.261	<.0001
PM_2.5_ (μg/m^3^)	2.512	2.149-2.936	<.0001	2.510	2.148-2.935	<.0001
PM_10_ (μg/m^3^)	1.518	1.354-1.701	<.0001	1.517	1.354-1.700	<.0001
NO (ppb)	1.922	1.653-2.235	<.0001	1.919	1.651-2.232	<.0001
NO_2_ (ppb)	1.625	1.405-1.880	<.0001	1.623	1.403-1.877	<.0001
NOx(ppb)	1.754	1.510-2.038	<.0001	1.752	1.508-2.035	<.0001
0-6 months						
SO_2_ (ppb)	1.971	1.725-2.253	<.0001	1.970	1.724-2.252	<.0001
PM_2.5_ (μg/m^3^)	2.605	2.216-3.063	<.0001	2.603	2.214-3.061	<.0001
PM_10_ (μg/m^3^)	1.535	1.368-1.723	<.0001	1.535	1.368-1.722	<.0001
NO (ppb)	1.918	1.649-2.230	<.0001	1.915	1.647-2.227	<.0001
NO_2_ (ppb)	1.630	1.406-1.890	<.0001	1.628	1.404-1.887	<.0001
NOx(ppb)	1.740	1.500-2.020	<.0001	1.738	1.497-2.017	<.0001
0-12 months						
SO_2_ (ppb)	2.042	1.779-2.343	<.0001	2.040	1.777-2.342	<.0001
PM_2.5_ (μg/m^3^)	2.538	2.172-2.966	<.0001	2.538	2.172-2.965	<.0001
PM_10_ (μg/m^3^)	1.559	1.394-1.743	<.0001	1.559	1.394-1.744	<.0001
NO (ppb)	1.952	1.676-2.275	<.0001	1.950	1.673-2.272	<.0001
NO_2_ (ppb)	1.645	1.422-1.903	<.0001	1.643	1.420-1.901	<.0001
NOx(ppb)	1.771	1.525-2.058	<.0001	1.769	1.523-2.056	<.0001

ORs (95% Cis) were estimated for per IQR increase in SO_2_, PM_2.5_, PM_10,_ NO, NO_2,_ and NO_X_

*Abbreviations*: SO_2_: sulphur dioxide; PM_2.5_: particulate matters with diameters at 2.5 micrometers and smaller; PM_10_: particulate matters with diameters at 10 micrometers and smaller; NO: nitrogen oxide; NO_2_: nitrogen dioxide; NO_X_: nitrogen oxides; OR: crude odds ratio; aOR: adjusted odds ratio

^a^Conditional logistic regressions were conducted controlling baseline demographic characteristics age, gender, allergic rhinitis, chronic sinusitis, and atopic dermatitis

**Fig. 2:**
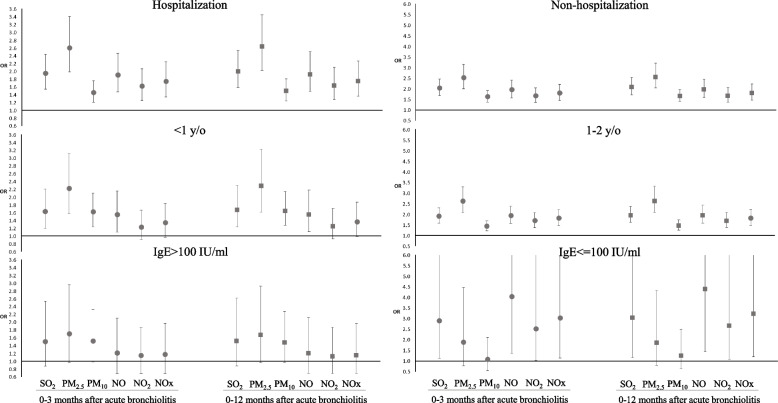
Subsequent exposure to ambient air pollutions at 0-3 and 0-12months and development of asthma among different subgroups

The sensitivity analysis depending on the acquired age, hospitalization and IgE level showed in Supplemental Tables 1, 2 and 3, separately. Figure 2 summarized the results from multiple conditional logistic regression models for the association between exposure to air pollutants and the risk of asthma in different subgroups with acute bronchiolitis in 0-3 and 0-12 months. Each specific air pollutants in each study periods all increases the risk of developing preschool asthma significantly in both groups of children whose first infected episode were age under one year old and age between one and two years old (Supplemental Table 1). When the infants had hospitalized acute bronchiolitis episode, the exposures to SO_2_, PM_2.5_, PM_10_, and NO during three, six, and twelve months after acute bronchiolitis are associated with developing preschool asthma. Exposure to PM_2.5_ had the highest and significant association with aORs (95% CIs) of 2.208 (1.568-3.108), 2.237 (1.576-3.175) and 2.286 (1.622-3.223) in the three, six, and twelve months after acute bronchiolitis, respectively. SO2 had also significant association with aORs (95% CIs) of 1.622 (1.197-2.197), 1.622 (1.200-2.193) and 1.679 (1.229-2.292) in the three, six, and twelve months after acute bronchiolitis, respectively. PM_10_ had also significant association with aORs (95% CIs) of 1.613 (1.240-2.097), 1.636 (1.251-2.140) and 1.650 (1.275-2.136) in the three, six, and twelve months after acute bronchiolitis, respectively. The association for NO was the lowest and significant association with aORs (95% CIs) of 1.541 (1.103-2.153), 1.525 (1.094-2.125) and 1.557 (1.112-2.181) in the three, six, and twelve months after acute bronchiolitis, respectively (Supplemental Table 2).

When the infants had IgE less or equal to 100 IU/ml, the exposures to SO_2_, NOx, NO_2_, and NO during three, six, and twelve months after acute bronchiolitis are associated with developing preschool asthma. Among the infants had IgE above 100 IU/ml, all pollutants in the study among three study periods are not associated with developing preschool asthma (Supplemental Table 3).

## Discussion

This cohort study found that exposures to ambient PM_2.5_, PM_10_, SO_2_, NO, NO_2_, and NOX during three, six and 12 months after acute bronchiolitis were associated with preschool asthma. With subgroup analysis, we found that only PM_2.5_, PM_10_, SO_2_, NO had the associations with preschool asthma during three study points in children who hospitalized due to acute bronchiolitis.

Limited studies explored the relationship between the air pollutions, past episode of bronchiolitis, and asthma. A prospective study in Korea with 1743 children demonstrated that the prevalence of asthma increased and lung function decreased in children with history of bronchiolitis who were exposed to higher CO、NO_2_、O_3_ in past five years [21]. However, the synergistic effect between air pollutant (2001-2005) and a history of bronchiolitis on the development of asthma during the two years follow up period (2005-2006) was not significant [21]. Another cohort study including with 920 infants acquired acute bronchiolitis before 1 years old in multiple major cities, U.S represented a possible synergistic air pollutions effects and asthma for high-risk child [22]. For these infants, proximity to major roads increases risk of both recurrent wheeze and asthma at three years and five years old, separately [22]. In our study, PM2.5 was the most significant component associated with the inception of preschool asthma. In animal experiments, exposure to PM2.5 can induce asthma by promoting inflammation in the airways and lungs through the induction of a Th1/Th2 inflammatory imbalance, activation of NF-κB, and the expression of miR-206 [23].

Even though strong association between acute bronchiolitis and inception of pediatric asthma, it is only about 30% of children with acute bronchiolitis would develop asthma later [24]. We hypothesized that the effect of exposure to air pollutions after bronchiolitis episode may increase the risk of developing asthma. Lower respiratory virus infection perturbing the immunological homeostasis in the mucosa dendritic cell populations even after viral clearance led to proinflammatory environment in the airway [4]. Increased responsiveness to concomitant exposure to aeroallergen with upregulation of FceR1 in infected lung tissues was found in mice model after virus infection [25]. Meanwhile, air pollutions can induce not only persistent inflammatory stated by disturbing epithelium immune system but directed damage from oxidative stress [26]. Therefore, basing on our result, we speculate that damaged respiratory epithelial cells led the child with previous acute bronchiolitis more vulnerable to the oxidation stress from air pollutants.

The reason why subsequent air pollutions were not associated with risk of developing asthma in children with high total IgE might be because in current study we only focus on the periods of exposure after bronchiolitis in developing preschool asthma. For them, exposure to ambient air pollutions in these periods might not further enhance allergic inflammation. A child with high total IgE might indicated allergic asthma, but not vice versa. For atopic children, previous allergen sensitization via atopic lesion-bone marrow axis and virus infections have great influence in asthma pathogenesis [4]. This result should be interpreted cautiously because the association between early exposure air pollutants, incidence and severity of bronchiolitis, and asthma had been confirmed [8]. According to our findings, primary prevention from ambient air pollutions post bronchiolitis episode should raise concerns for all the children whether had atopic or not. However, we needed more studies on how acute bronchiolitis interacted with air pollutants in developing pediatric asthma such as nasal epigenic microRNAs, reported as potential biomarker for treatment [27].

The present study has important strengths. We first explored the role of PM_10_, PM_2.5_, SO_2_ in the association between air pollutant exposure after acute bronchiolitis and preschool asthma. We acknowledge some limitations in our study. First, although we tried to use high IgE level, early infection age, and hospitalization to indicated children with atopic disease and severe bronchiolitis, we were unable to identify important covariates associated with developing asthma in child with severe bronchiolitis, such as virus subtype, family history of atopic disease, maternal asthma, and socioeconomic status which might alert the interpretations of our result [24, 28]. Second, the effect of the residential migration and indoor air pollution were underestimated which have been proven associated with childhood asthma. Third, as high IgE level was strongly associated with developing asthma among atopic children, there were not all the cases in our study with total IgE data.

In conclusion, ambient air pollutants till 12 months after acute bronchiolitis was still associated with developing preschool asthma. Our study raised the concern of the pollutants with acute bronchiolitis which was less attention in the current study trend. Further studies should investigate the influence with specific virus subtype and other biomarker for more precise primary prevention strategy.

### Supplementary Information

**Additional file 1:** **Supplemental Table 1.** Associations between subsequent exposure to ambient air pollutants and preschool asthma in infants acquired infection younger than 1 year old and between 1 and 2 years old in terms of odds ratio (OR) and 95% CI.

**Additional file 2:** **Supplemental Table 2.** Associations between subsequent exposure to ambient air pollutants and preschool asthma in infants with hospitalization and none hospitalization in terms of odds ratio (OR) and 95% CI.

** Additional file 3: Supplementary Table 3.** Associations between subsequent exposure to ambient air pollutants and preschool asthma in infants with total IgE level in terms of odds ratio (OR) and 95% CI ( *n*  = 793).

## Data Availability

The datasets generated during and/or analyzing during the current study are not publicly available due to the use from the initial informed consent.

## Abbreviations

aORs: Adjusted ORs
CIs: Confidence intervals
EMRs: Electronic medical records
ICD-9-CM: International Classification of Diseases, Ninth Revision, Clinical Modification
IQR: Interquartile range
KMUHRD: Kaohsiung Medical University Hospital Research Database
NO: Nitric oxide
NO_2_: Nitrogen dioxide
NO_X_: Nitrogen oxides
ORs: Odds ratios
PM_2.5_: Particulate matter of 2.5 mm in diameter
PM_10_: Particulate matter of 10 mm in diameter
PSM: Propensity score matching
