# Supplementary material for: Air pollution after acute bronchiolitis is a risk factor for preschool asthma: a nested case-control study
Source: Environ Health. 2023 Dec 4;22:83. doi: 10.1186/s12940-023-01035-1 (PMC10694905; doi:10.1186/s12940-023-01035-1)
Supplement: Supplementary file 1 — Additional file 1: Supplemental Table 1. Associations between subsequent exposure to ambient air pollutants and preschool asthma in infants acquired infection younger than 1 year old and between 1 and 2 years old in terms of odds ratio (OR) and 95% CI. [file 12940_2023_1035_MOESM1_ESM.docx]

**Supplemental Table 1.** Associations between subsequent exposure to ambient air pollutants and preschool asthma in infants acquired infection younger than 1 year old and between 1 and 2 years old in terms of odds ratio (OR) and 95% CI

|  | | | | |  | |  | |  | |  |  | |  |
| --- | --- | --- | --- | --- | --- | --- | --- | --- | --- | --- | --- | --- | --- | --- |
|  | Crude OR | | 95%CI | | p-value | | Adjusted OR | | 95%CI | | p-value |  | |  |
| **First bronchiolitis episode younger than 1 year old (n=1149)** | | | | | | |  | |  | |  |  | |  |
| 0-3 months |  | |  | |  | |  | |  | |  |  | |  |
| SO_2_ (ppb) | 1.941 | | 1.544-2.439 | | <.0001 | | 1.940 | | 1.542-2.440 | | <.0001 |  | |  |
| PM_2.5_ (μg/m^3^) | 2.598 | | 1.982-3.406 | | <.0001 | | 2.598 | | 1.982-3.406 | | <.0001 |  | |  |
| PM_10_ (μg/m^3^) | 1.453 | | 1.202-1.755 | | 0.000 | | 1.451 | | 1.201-1.754 | | 0.000 |  | |  |
| NO (ppb) | 1.902 | | 1.471-2.459 | | <.0001 | | 1.900 | | 1.469-2.457 | | <.0001 |  | |  |
| NO_2_ (ppb) | 1.612 | | 1.258-2.066 | | 0.000 | | 1.610 | | 1.256-2.064 | | 0.000 |  | |  |
| NOx(ppb) | 1.738 | | 1.346-2.243 | | <.0001 | | 1.735 | | 1.343-2.240 | | <.0001 |  | |  |
| 0-6 months |  | |  | |  | |  | |  | |  |  | |  |
| SO_2_ (ppb) | 1.934 | | 1.541-2.427 | | <.0001 | | 1.934 | | 1.540-2.428 | | <.0001 |  | |  |
| PM_2.5_ (μg/m^3^) | 2.727 | | 2.065-3.603 | | <.0001 | | 2.729 | | 2.066-3.606 | | <.0001 |  | |  |
| PM_10_ (μg/m^3^) | 1.485 | | 1.226-1.797 | | <.0001 | | 1.483 | | 1.225-1.795 | | <.0001 |  | |  |
| NO (ppb) | 1.900 | | 1.469-2.455 | | <.0001 | | 1.897 | | 1.467-2.454 | | <.0001 |  | |  |
| NO_2_ (ppb) | 1.624 | | 1.262-2.089 | | 0.000 | | 1.621 | | 1.260-2.086 | | 0.000 |  | |  |
| NOx(ppb) | 1.729 | | 1.342-2.228 | | <.0001 | | 1.727 | | 1.340-2.225 | | <.0001 |  | |  |
| 0-12 months |  | |  | |  | |  | |  | |  |  | |  |
| SO_2_ (ppb) | 2.004 | | 1.584-2.534 | | <.0001 | | 2.003 | | 1.583-2.534 | | <.0001 |  | |  |
| PM_2.5_ (μg/m^3^) | 2.640 | | 2.019-3.452 | | <.0001 | | 2.639 | | 2.018-3.451 | | <.0001 |  | |  |
| PM_10_ (μg/m^3^) | 1.500 | | 1.243-1.809 | | <.0001 | | 1.498 | | 1.242-1.807 | | <.0001 |  | |  |
| NO (ppb) | 1.929 | | 1.487-2.504 | | <.0001 | | 1.927 | | 1.484-2.502 | | <.0001 |  | |  |
| NO_2_ (ppb) | 1.638 | | 1.277-2.099 | | <.0001 | | 1.635 | | 1.275-2.097 | | 0.000 |  | |  |
| NOx(ppb) | 1.757 | | 1.361-2.268 | | <.0001 | | 1.755 | | 1.359-2.266 | | <.0001 |  | |  |
| **First bronchiolitis episode between 1-2 years old (n=1488)** | | | | | | |  | |  | |  |  | |  |
| 0-3 months |  | |  | |  | |  | |  | |  |  | |  |
| SO_2_ (ppb) | 2.025 | | 1.668-2.457 | | <.0001 | | 2.025 | | 1.668-2.457 | | <.0001 |  | |  |
| PM_2.5_ (μg/m^3^) | 2.515 | | 2.006-3.153 | | <.0001 | | 2.514 | | 2.005-3.152 | | <.0001 |  | |  |
| PM_10_ (μg/m^3^) | 1.619 | | 1.365-1.920 | | <.0001 | | 1.620 | | 1.364-1.923 | | <.0001 |  | |  |
| NO (ppb) | 1.949 | | 1.574-2.413 | | <.0001 | | 1.948 | | 1.573-2.411 | | <.0001 |  | |  |
| NO_2_ (ppb) | 1.665 | | 1.360-2.039 | | <.0001 | | 1.664 | | 1.359-2.037 | | <.0001 |  | |  |
| NOx(ppb) | 1.790 | | 1.451-2.208 | | <.0001 | | 1.788 | | 1.449-2.205 | | <.0001 |  | |  |
| 0-6 months |  | |  | |  | |  | |  | |  |  | |  |
| SO_2_ (ppb) | 2.015 | | 1.662-2.443 | | <.0001 | | 2.015 | | 1.662-2.444 | | <.0001 |  | |  |
| PM_2.5_ (μg/m^3^) | 2.552 | | 2.017-3.229 | | <.0001 | | 2.550 | | 2.015-3.227 | | <.0001 |  | |  |
| PM_10_ (μg/m^3^) | 1.605 | | 1.352-1.906 | | <.0001 | | 1.605 | | 1.351-1.908 | | <.0001 |  | |  |
| NO (ppb) | 1.935 | | 1.563-2.396 | | <.0001 | | 1.934 | | 1.562-2.395 | | <.0001 |  | |  |
| NO_2_ (ppb) | 1.655 | | 1.347-2.034 | | <.0001 | | 1.654 | | 1.346-2.032 | | <.0001 |  | |  |
| NOx(ppb) | 1.762 | | 1.430-2.170 | | <.0001 | | 1.760 | | 1.429-2.169 | | <.0001 |  | |  |
| 0-12 months | |  | |  | |  | |  | |  |  |  |  |  |
| SO_2_ (ppb) | 2.087 | | 1.711-2.545 | | <.0001 | | 2.087 | | 1.711-2.545 | | <.0001 |  | |  |
| PM_2.5_ (μg/m^3^) | 2.562 | | 2.042-3.215 | | <.0001 | | 2.562 | | 2.041-3.216 | | <.0001 |  | |  |
| PM_10_ (μg/m^3^) | 1.671 | | 1.414-1.973 | | <.0001 | | 1.672 | | 1.414-1.977 | | <.0001 |  | |  |
| NO (ppb) | 1.977 | | 1.593-2.455 | | <.0001 | | 1.976 | | 1.592-2.453 | | <.0001 |  | |  |
| NO_2_ (ppb) | 1.685 | | 1.376-2.063 | | <.0001 | | 1.683 | | 1.375-2.061 | | <.0001 |  | |  |
| NOx(ppb) | 1.806 | | 1.464-2.228 | | <.0001 | | 1.805 | | 1.463-2.226 | | <.0001 |  | |  |
| **Notes:** *Conditional logistic regressions were conducted controlling baseline demographic characteristics age, gender, allergic rhinitis, chronic sinusitis, and atopic dermatitis.  ORs (95% CIs) were estimated for per IQR increase in SO_2_, PM_2.5_, PM_10,_ NO, NO_2,_ and NO_X._  **Abbreviations:**  SO_2_=sulphur dioxide; PM_2.5_=particulate matters with diameters at 2.5 micrometers and smaller; PM_10=_ particulate matters with diameters at 10 micrometers and smaller; NO = nitrogen oxide (NO); NO_2_=nitrogen dioxide; NO_X_ = nitrogen oxides; OR=crude odds ratio; aOR=adjusted odds ratio  IQR= interquartile range | | | | | | | | | | | | |  |  |
