# Supplementary material for: Air pollution after acute bronchiolitis is a risk factor for preschool asthma: a nested case-control study
Source: Environ Health. 2023 Dec 4;22:83. doi: 10.1186/s12940-023-01035-1 (PMC10694905; doi:10.1186/s12940-023-01035-1)
Supplement: Supplementary file 2 — Additional file 2: Supplemental Table 2. Associations between subsequent exposure to ambient air pollutants and preschool asthma in infants with hospitalization and none hospitalization in terms of odds ratio (OR) and 95% CI. [file 12940_2023_1035_MOESM2_ESM.docx]

**Supplemental Table 2.** Associations between subsequent exposure to ambient air pollutants and preschool asthma in infants with hospitalization and none hospitalization in terms of odds ratio (OR) and 95% CI

|  | | | | |  | |  | |  | |  |  | |  |
| --- | --- | --- | --- | --- | --- | --- | --- | --- | --- | --- | --- | --- | --- | --- |
|  | Crude OR | | 95%CI | | p-value | | Adjusted OR | | 95%CI | | p-value |  | |  |
| **Hospitalization for first bronchiolitis (n=839)** | | | | |  | |  | |  | |  |  | |  |
| 0-3 months |  | |  | |  | |  | |  | |  |  | |  |
| SO_2_ (ppb) | 1.616 | | 1.193-2.189 | | 0.002 | | 1.622 | | 1.197-2.197 | | 0.002 |  | |  |
| PM_2.5_ (μg/m^3^) | 2.226 | | 1.585-3.127 | | <.0001 | | 2.208 | | 1.568-3.108 | | <.0001 |  | |  |
| PM_10_ (μg/m^3^) | 1.628 | | 1.256-2.111 | | 0.000 | | 1.613 | | 1.240-2.097 | | 0.000 |  | |  |
| NO (ppb) | 1.529 | | 1.096-2.133 | | 0.013 | | 1.541 | | 1.103-2.153 | | 0.011 |  | |  |
| NO_2_ (ppb) | 1.210 | | 0.891-1.643 | | 0.222 | | 1.225 | | 0.901-1.666 | | 0.196 |  | |  |
| NOx(ppb) | 1.321 | | 0.958-1.821 | | 0.089 | | 1.336 | | 0.968-1.844 | | 0.078 |  | |  |
| 0-6 months |  | |  | |  | |  | |  | |  |  | |  |
| SO_2_ (ppb) | 1.616 | | 1.195-2.184 | | 0.002 | | 1.622 | | 1.200-2.193 | | 0.002 |  | |  |
| PM_2.5_ (μg/m^3^) | 2.256 | | 1.592-3.197 | | <.0001 | | 2.237 | | 1.576-3.175 | | <.0001 |  | |  |
| PM_10_ (μg/m^3^) | 1.653 | | 1.267-2.157 | | 0.000 | | 1.636 | | 1.251-2.140 | | 0.000 |  | |  |
| NO (ppb) | 1.511 | | 1.085-2.102 | | 0.014 | | 1.525 | | 1.094-2.125 | | 0.013 |  | |  |
| NO_2_ (ppb) | 1.200 | | 0.881-1.635 | | 0.248 | | 1.217 | | 0.892-1.661 | | 0.215 |  | |  |
| NOx(ppb) | 1.303 | | 0.950-1.788 | | 0.101 | | 1.320 | | 0.961-1.814 | | 0.087 |  | |  |
| 0-12 months |  | |  | |  | |  | |  | |  |  | |  |
| SO_2_ (ppb) | 1.673 | | 1.225-2.284 | | 0.001 | | 1.679 | | 1.229-2.292 | | 0.001 |  | |  |
| PM_2.5_ (μg/m^3^) | 2.303 | | 1.637-3.240 | | <.0001 | | 2.286 | | 1.622-3.223 | | <.0001 |  | |  |
| PM_10_ (μg/m^3^) | 1.665 | | 1.290-2.148 | | <.0001 | | 1.650 | | 1.275-2.136 | | 0.000 |  | |  |
| NO (ppb) | 1.545 | | 1.105-2.160 | | 0.011 | | 1.557 | | 1.112-2.181 | | 0.010 |  | |  |
| NO_2_ (ppb) | 1.241 | | 0.915-1.683 | | 0.165 | | 1.256 | | 0.925-1.706 | | 0.144 |  | |  |
| NOx(ppb) | 1.345 | | 0.978-1.850 | | 0.068 | | 1.360 | | 0.988-1.874 | | 0.060 |  | |  |
| **None Hospitalization for first bronchiolitis (n= 1798)** | | | | |  | |  | |  | |  |  | |  |
| 0-3 months |  | |  | |  | |  | |  | |  |  | |  |
| SO_2_ (ppb) | 1.909 | | 1.585-2.298 | | <.0001 | | 1.908 | | 1.585-2.297 | | <.0001 |  | |  |
| PM_2.5_ (μg/m^3^) | 2.610 | | 2.078-3.280 | | <.0001 | | 2.618 | | 2.083-3.292 | | <.0001 |  | |  |
| PM_10_ (μg/m^3^) | 1.426 | | 1.208-1.683 | | <.0001 | | 1.426 | | 1.208-1.683 | | <.0001 |  | |  |
| NO (ppb) | 1.929 | | 1.562-2.382 | | <.0001 | | 1.930 | | 1.562-2.384 | | <.0001 |  | |  |
| NO_2_ (ppb) | 1.690 | | 1.376-2.077 | | <.0001 | | 1.691 | | 1.376-2.077 | | <.0001 |  | |  |
| NOx(ppb) | 1.804 | | 1.460-2.228 | | <.0001 | | 1.805 | | 1.461-2.229 | | <.0001 |  | |  |
| 0-6 months |  | |  | |  | |  | |  | |  |  | |  |
| SO_2_ (ppb) | 1.907 | | 1.585-2.293 | | <.0001 | | 1.907 | | 1.585-2.293 | | <.0001 |  | |  |
| PM_2.5_ (μg/m^3^) | 2.730 | | 2.155-3.458 | | <.0001 | | 2.739 | | 2.161-3.472 | | <.0001 |  | |  |
| PM_10_ (μg/m^3^) | 1.459 | | 1.233-1.725 | | <.0001 | | 1.459 | | 1.233-1.727 | | <.0001 |  | |  |
| NO (ppb) | 1.937 | | 1.567-2.394 | | <.0001 | | 1.938 | | 1.568-2.395 | | <.0001 |  | |  |
| NO_2_ (ppb) | 1.710 | | 1.386-2.111 | | <.0001 | | 1.710 | | 1.385-2.111 | | <.0001 |  | |  |
| NOx(ppb) | 1.804 | | 1.461-2.227 | | <.0001 | | 1.804 | | 1.461-2.227 | | <.0001 |  | |  |
| 0-12 months | |  | |  | |  | |  | |  |  |  |  |  |
| SO_2_ (ppb) | 1.960 | | 1.621-2.372 | | <.0001 | | 1.960 | | 1.621-2.371 | | <.0001 |  | |  |
| PM_2.5_ (μg/m^3^) | 2.626 | | 2.090-3.298 | | <.0001 | | 2.634 | | 2.096-3.311 | | <.0001 |  | |  |
| PM_10_ (μg/m^3^) | 1.479 | | 1.255-1.743 | | <.0001 | | 1.479 | | 1.255-1.743 | | <.0001 |  | |  |
| NO (ppb) | 1.960 | | 1.582-2.429 | | <.0001 | | 1.961 | | 1.583-2.431 | | <.0001 |  | |  |
| NO_2_ (ppb) | 1.700 | | 1.383-2.090 | | <.0001 | | 1.701 | | 1.383-2.091 | | <.0001 |  | |  |
| NOx(ppb) | 1.815 | | 1.469-2.243 | | <.0001 | | 1.815 | | 1.469-2.244 | | <.0001 |  | |  |
| **Notes:** *Conditional logistic regressions were conducted controlling baseline demographic characteristics age, gender, allergic rhinitis, chronic sinusitis, and atopic dermatitis.  ORs (95% CIs) were estimated for per IQR increase in SO_2_, PM_2.5_, PM_10,_ NO, NO_2,_ and NO_X._  **Abbreviations:**  SO_2_=sulphur dioxide; PM_2.5_=particulate matters with diameters at 2.5 micrometers and smaller; PM_10=_ particulate matters with diameters at 10 micrometers and smaller; NO = nitrogen oxide (NO); NO_2_=nitrogen dioxide; NO_X_ = nitrogen oxides; OR=crude odds ratio; aOR=adjusted odds ratio  IQR= interquartile range | | | | | | | | | | | | |  |  |
