# Supplementary material for: Air pollution after acute bronchiolitis is a risk factor for preschool asthma: a nested case-control study
Source: Environ Health. 2023 Dec 4;22:83. doi: 10.1186/s12940-023-01035-1 (PMC10694905; doi:10.1186/s12940-023-01035-1)
Supplement: Supplementary file 3 — Additional file 3: Supplementary Table 3. Associations between subsequent exposure to ambient air pollutants and preschool asthma in infants with total IgE level in terms of odds ratio (OR) and 95% CI ( n = 793). [file 12940_2023_1035_MOESM3_ESM.docx]

**Supplementary Table 3.** Associations between subsequent exposure to ambient air pollutants and preschool asthma in infants with total IgE level in terms of odds ratio (OR) and 95% CI (n= 793)

|  | | | | |  | |  | |  | |  |  |  |
| --- | --- | --- | --- | --- | --- | --- | --- | --- | --- | --- | --- | --- | --- |
|  | Crude OR | | 95%CI | | p-value | | Adjusted OR | | 95%CI | | p-value |  |  |
| **IgE > 100 (n=462)** | | | | |  | |  | |  | |  |  |  |
| 0-3 months |  | |  | |  | |  | |  | |  |  |  |
| SO_2_ (ppb) | 1.481 | | 0.869-2.525 | | 0.149 | | 1.488 | | 0.872-2.537 | | 0.145 |  |  |
| PM_2.5_ (μg/m^3^) | 1.637 | | 0.952-2.816 | | 0.075 | | 1.691 | | 0.966-2.960 | | 0.066 |  |  |
| PM_10_ (μg/m^3^) | 1.502 | | 0.976-2.310 | | 0.065 | | 1.504 | | 0.977-2.317 | | 0.064 |  |  |
| NO (ppb) | 1.205 | | 0.691-2.100 | | 0.511 | | 1.205 | | 0.691-2.099 | | 0.511 |  |  |
| NO_2_ (ppb) | 1.140 | | 0.695-1.869 | | 0.604 | | 1.139 | | 0.694-1.868 | | 0.606 |  |  |
| NOx(ppb) | 1.166 | | 0.691-1.966 | | 0.566 | | 1.165 | | 0.690-1.965 | | 0.568 |  |  |
| 0-6 months |  | |  | |  | |  | |  | |  |  |  |
| SO_2_ (ppb) | 1.454 | | 0.859-2.460 | | 0.163 | | 1.461 | | 0.863-2.473 | | 0.158 |  |  |
| PM_2.5_ (μg/m^3^) | 1.629 | | 0.934-2.840 | | 0.086 | | 1.688 | | 0.951-2.997 | | 0.074 |  |  |
| PM_10_ (μg/m^3^) | 1.518 | | 0.969-2.377 | | 0.068 | | 1.522 | | 0.971-2.385 | | 0.067 |  |  |
| NO (ppb) | 1.201 | | 0.691-2.089 | | 0.516 | | 1.202 | | 0.691-2.089 | | 0.515 |  |  |
| NO_2_ (ppb) | 1.127 | | 0.682-1.864 | | 0.640 | | 1.127 | | 0.681-1.863 | | 0.643 |  |  |
| NOx(ppb) | 1.153 | | 0.686-1.936 | | 0.591 | | 1.152 | | 0.686-1.935 | | 0.592 |  |  |
| 0-12 months |  | |  | |  | |  | |  | |  |  |  |
| SO_2_ (ppb) | 1.506 | | 0.873-2.598 | | 0.141 | | 1.513 | | 0.877-2.612 | | 0.137 |  |  |
| PM_2.5_ (μg/m^3^) | 1.621 | | 0.947-2.774 | | 0.078 | | 1.676 | | 0.961-2.922 | | 0.069 |  |  |
| PM_10_ (μg/m^3^) | 1.477 | | 0.959-2.273 | | 0.077 | | 1.479 | | 0.960-2.279 | | 0.076 |  |  |
| NO (ppb) | 1.207 | | 0.687-2.120 | | 0.513 | | 1.207 | | 0.687-2.119 | | 0.513 |  |  |
| NO_2_ (ppb) | 1.127 | | 0.684-1.859 | | 0.638 | | 1.127 | | 0.683-1.857 | | 0.641 |  |  |
| NOx(ppb) | 1.156 | | 0.683-1.958 | | 0.589 | | 1.156 | | 0.683-1.957 | | 0.590 |  |  |
| **IgE≦100 (n=331)** | | | | |  | |  | |  | |  |  |  |
| 0-3 months |  | |  | |  | |  | |  | |  |  |  |
| SO_2_ (ppb) | 2.736 | | 1.071-6.991 | | 0.036 | | 2.882 | | 1.120-7.419 | | 0.028 |  |  |
| PM_2.5_ (μg/m^3^) | 1.840 | | 0.764-4.433 | | 0.174 | | 1.864 | | 0.777-4.471 | | 0.163 |  |  |
| PM_10_ (μg/m^3^) | 1.089 | | 0.565-2.099 | | 0.800 | | 1.065 | | 0.538-2.107 | | 0.857 |  |  |
| NO (ppb) | 3.676 | | 1.281-10.547 | | 0.016 | | 4.026 | | 1.359-11.923 | | 0.012 |  |  |
| NO_2_ (ppb) | 2.351 | | 0.995-5.560 | | 0.052 | | 2.499 | | 1.025-6.090 | | 0.044 |  |  |
| NOx(ppb) | 2.800 | | 1.087-7.215 | | 0.033 | | 3.013 | | 1.135-7.997 | | 0.027 |  |  |
| 0-6 months |  | |  | |  | |  | |  | |  |  |  |
| SO_2_ (ppb) | 2.646 | | 1.065-6.576 | | 0.036 | | 2.811 | | 1.115-7.088 | | 0.029 |  |  |
| PM_2.5_ (μg/m^3^) | 1.692 | | 0.718-3.989 | | 0.230 | | 1.735 | | 0.734-4.104 | | 0.210 |  |  |
| PM_10_ (μg/m^3^) | 1.058 | | 0.530-2.112 | | 0.873 | | 1.035 | | 0.509-2.106 | | 0.925 |  |  |
| NO (ppb) | 3.617 | | 1.282-10.209 | | 0.015 | | 3.976 | | 1.360-11.620 | | 0.012 |  |  |
| NO_2_ (ppb) | 2.270 | | 0.978-5.272 | | 0.057 | | 2.440 | | 1.010-5.894 | | 0.047 |  |  |
| NOx(ppb) | 2.662 | | 1.072-6.612 | | 0.035 | | 2.890 | | 1.122-7.445 | | 0.028 |  |  |
| 0-12 months | |  | |  | |  | |  | |  |  |  |  |
| SO_2_ (ppb) | 2.869 | | 1.094-7.529 | | 0.032 | | 3.042 | | 1.149-8.055 | | 0.025 |  |  |
| PM_2.5_ (μg/m^3^) | 1.845 | | 0.789-4.316 | | 0.158 | | 1.860 | | 0.800-4.326 | | 0.150 |  |  |
| PM_10_ (μg/m^3^) | 1.268 | | 0.653-2.459 | | 0.483 | | 1.255 | | 0.631-2.494 | | 0.518 |  |  |
| NO (ppb) | 4.029 | | 1.353-11.999 | | 0.012 | | 4.394 | | 1.436-13.444 | | 0.010 |  |  |
| NO_2_ (ppb) | 2.494 | | 1.032-6.027 | | 0.042 | | 2.671 | | 1.070-6.666 | | 0.035 |  |  |
| NOx(ppb) | 2.989 | | 1.135-7.876 | | 0.027 | | 3.227 | | 1.191-8.744 | | 0.021 |  |  |
| **Notes:** *Conditional logistic regressions were conducted controlling baseline demographic characteristics age, gender, allergic rhinitis, chronic sinusitis, and atopic dermatitis.  ORs (95% CIs) were estimated for per IQR increase in SO_2_, PM_2.5_, PM_10,_ NO, NO_2,_ and NO_X._  **Abbreviations:**  SO_2_=sulphur dioxide; PM_2.5_=particulate matters with diameters at 2.5 micrometers and smaller; PM_10=_ particulate matters with diameters at 10 micrometers and smaller; NO = nitrogen oxide (NO); NO_2_=nitrogen dioxide; NO_X_ = nitrogen oxides; OR=crude odds ratio; aOR=adjusted odds ratio  IQR= interquartile range | | | | | | | | | | | | |  |
